# MARCH5 requires MTCH2 to coordinate proteasomal turnover of the MCL1:NOXA complex

**DOI:** 10.1038/s41418-020-0517-0

**Published:** 2020-02-24

**Authors:** Tirta Mario Djajawi, Lei Liu, Jia-nan Gong, Allan Shuai Huang, Ming-jie Luo, Zhen Xu, Toru Okamoto, Melissa J. Call, David C. S. Huang, Mark F. van Delft

**Affiliations:** 1 The Walter and Eliza Hall Institute of Medical Research, Parkville, VIC Australia; 2 Department of Medical Biology, University of Melbourne, Parkville, VIC Australia; 3 School of Medicine, Tsinghua University, Beijing, China; 4 Institute for Advanced Co-Creation Studies, Research Institute for Microbial Diseases, Osaka University, Osaka, Japan; 5 Present Address: Institute for Brain Research and Rehabilitation, Guangdong Key Laboratory of Mental Health and Cognitive Science, Center for Studies of Psychological Application, South China Normal University, Guangzhou, China

**Keywords:** Ubiquitylation, Ubiquitylation, Ubiquitin ligases

## Abstract

MCL1, a BCL2 relative, is critical for the survival of many cells. Its turnover is often tightly controlled through both ubiquitin-dependent and -independent mechanisms of proteasomal degradation. Several cell stress signals, including DNA damage and cell cycle arrest, are known to elicit distinct E3 ligases to ubiquitinate and degrade MCL1. Another trigger that drives MCL1 degradation is engagement by NOXA, one of its BH3-only protein ligands, but the mechanism responsible has remained unclear. From an unbiased genome-wide CRISPR-Cas9 screen, we discovered that the ubiquitin E3 ligase MARCH5, the ubiquitin E2 conjugating enzyme UBE2K, and the mitochondrial outer membrane protein MTCH2 co-operate to mark MCL1 for degradation by the proteasome—specifically when MCL1 is engaged by NOXA. This mechanism of degradation also required the MCL1 transmembrane domain and distinct MCL1 lysine residues to proceed, suggesting that the components likely act on the MCL1:NOXA complex by associating with it in a specific orientation within the mitochondrial outer membrane. MTCH2 has not previously been reported to regulate protein stability, but is known to influence the mitochondrial localization of certain key apoptosis regulators and to impact metabolism. We have now pinpointed an essential but previously unappreciated role for MTCH2 in turnover of the MCL1:NOXA complex by MARCH5, further strengthening its links to BCL2-regulated apoptosis.

## Introduction

Intrinsic apoptosis is a fundamental process that must be carefully balanced to maintain tissue homeostasis and preserve the well-being of multicellular organisms. In vertebrates, the most critical regulators of apoptosis are the members of the BCL2 protein family, which include BCL2 and its prosurvival relatives (MCL1, BCLxL, BCLW, and A1), the proapoptotic BH3-only proteins (NOXA, BIM, PUMA, BAD, and BID) and the effector proteins BAX and BAK. The balance of interactions between these proteins dictates whether BAX and BAK become activated to permeabilize the mitochondrial outer membrane and cause apoptosis [1, 2]. Precise control of apoptosis is achieved by regulating BCL2 proteins in multiple ways including through posttranslational modifications that impact their stability, localization, and/or propensity to interact [3, 4].

MCL1 is a prosurvival relative of BCL2 and has been implicated in tumorigenesis and the resistance of cancer cells to therapy [5]. A distinct feature of MCL1 is its rapid turnover, which has been proposed as a mechanism to drive apoptosis in various settings [6–11]. Degradation of MCL1 provoked by the ubiquitin E3 ligases MULE and FBW7, for example, has been shown to facilitate the clearance of unfit cells following DNA damage and cell cycle arrest, respectively [12–14].

MCL1 is also rapidly degraded when it forms a complex with the BH3-only protein NOXA, but the mechanism responsible has remained unclear [15–17]. Intriguingly, NOXA is the only BH3-only protein that targets MCL1 for degradation. Most others that interact with MCL1, including BIM, instead enhance MCL1 stability [17, 18]. The structures of MCL1 in complex with the BH3 domains of either NOXA or BIM reveal no significant alterations to MCL1 conformation [16], suggesting that elements from both MCL1 and NOXA may combine to promote rapid degradation of the complex.

To interrogate the molecular pathway by which NOXA causes MCL1 to be degraded, we performed an unbiased loss-of-function genome-wide CRISPR-Cas9 screen. Here, we identify the mitochondrial ubiquitin E3 ligase MARCH5, the ubiquitin E2 conjugating enzyme UBE2K, and the mitochondrial protein MTCH2 as regulators that cooperate to target the MCL1:NOXA complex for degradation by the proteasome. MARCH5 has previously been reported to ubiquitinate MCL1 and drive its degradation [19, 20], but the requirements for MTCH2 and NOXA in this process have not been appreciated. Our observations indicate that this pathway contributes to MCL1 turnover in diverse cells and influences MCL1 steady-state levels in those that express significant amounts of NOXA.

## Methods and materials

### Gene expression using retroviral and lentiviral vectors

Retrovirus constructs encoding HA-tagged NOXA, NOXA3E, and tBID have been described [21, 22]. 3xMYC (3xMEQKLISEEDLNE)-tagged wild-type (WT) MARCH5 or MARCH5^C65/68S^ and FLAG (MDYKDDDDKGS)-tagged MTCH2 were synthesized as gene fragments by Integrated DNA Technologies (IDT) and subcloned into pMSCV-IRES-puro retroviral expression vector. MCL1^K175R^, MCL1^K178R^, MCL1^K175R, K178R^ and MCL1^14KR^ were generated by PCR using overlap-extension site-directed mutagenesis and subcloned into pMSCV-IRES-hygro. PCR fragments for MCL1 and MCL1^K117R, K175R, K178R^ were generated in the same way and subcloned in frame with an N-terminal GFP tag in pMSCV-IRES-hygro to facilitate FACS-based screening. MCL1^BCL2TM^ was synthesized as a gene fragment by IDT and subcloned in frame to replace the C-terminus of FLAG-tagged MCL1 in pMSCV-IRES-GFP. All constructs were confirmed by Sanger sequencing. FUCas9Cherry lentiviral construct was obtained from Marco Herold [23].

Viral particles were produced in packaging cells (Phoenix-ECO, Phoenix-AMPHO, 293T) and used to spin-infect mammalian cells as described [21]. Infected cells were cultured overnight, and then selected for the expression of antibiotic-resistance markers (hygromycin or puromycin) or sorted on the expression of fluorescent proteins (GFP, mCherry, or BFP).

### Cell culture

Mouse Embryonic Fibroblasts (MEFs) were cultured in DMEM supplemented with 10% (v/v) fetal calf serum (FCS), 100 μM asparagine and 50 μM of β-mercaptoethanol (β-Met). HELA cells were cultured in DMEM media supplemented with 10% (v/v) FCS. T47D, MCF7, MDA-MB-231, NCI-H522, HOP62, HOP92, IGROV1, RXF393, 786-0, TK10, HCT116, PC3, MALME3M, LOXIMVI, KMS-28-BM, RPMI8226, KMS-12-PE, MOLT4, RS4;11, and SR cells were cultured in RPMI1640 supplemented with 10% (v/v) FCS. All cells were cultured humidified incubators maintained at 37 °C and 10% CO_2_ for DMEM media or 5% CO_2_ for RPMI media.

All mouse fibroblast cell lines were generated from E13.5 embryos derived from mice on an inbred C57BL/6 background, and transformed with SV40 large T antigen. *BAX*^*−/−*^ *BAK*^*−/−*^ HELA cells were generated from HELA CCL2 and deletion of BAX and BAK was confirmed by sequencing. All other cell lines were obtained from ATCC, DSMZ, or JCRB cell line repositories. All cell lines were confirmed to be mycoplasma negative using the MycoAlert detection assay (Lonza).

Where indicated, cells were cultured with the following compounds: doxycycline (Sigma), nocodazole (Sigma), cycloheximide (Sigma), QVD-OPh (MP Biomedicals), MG132 (Sigma).

### Immunoblotting, subcellular fractionation, and immunoprecipitation

Whole cell protein extracts were prepared using ONYX lysis buffer (20 mM Tris-pH 7.4, 135 mM NaCl, 1.5 mM MgCl_2_, 1 mM EGTA, 10% (v/v) glycerol, 1% (v/v) Triton X-100, complete protease inhibitors (0.5 μg/ml)). All immunoblotting and immunoprecipitation experiments were performed using whole cell protein extracts unless otherwise specified. For subcellular fractionation, cells were first permeabilized with digitonin lysis buffer (20 mM Hepes, 100 mM KCl, 5 mM MgCl_2_, 1 mM EDTA, 1 mM EGTA and 250 mM sucrose, 0.025% (w/v) digitonin, complete protease inhibitors (0.5 μg/ml)) to extract cytosolic proteins before cell pellets were lysed in an equal volume of ONYX lysis buffer to extract membrane proteins. For immunoprecipitation, bait proteins were captured with monoclonal antibodies to MCL1 (19C4) or HA (3F10) and Protein G Sepharose beads.

In all cases, proteins were separated by SDS-PAGE, transferred into nitrocellulose membrane using the iBLOT dry blotting system (Invitrogen) and detected by immunoblotting. Monoclonal antibodies to MCL1 (19C4), FLAG (9H1), MYC (9E10), BIM (3C5), BAX (21C10 & 49F9), BAK (7D10), BCL2 (BCL-2-100), and HSP70 (N6; gift of W. Welch and R. Anderson) were produced within the WEHI antibody facility. The following antibodies were obtained from commercial sources: NOXA (114C307; Novus Biologicals), MCL1 (600-401-394S; Rockland), ACTIN (I-19, Santa Cruz Biotechnology), HA (3F10; Roche Applied Science), MARCH5 (ab174959; Abcam), UBE2K (EP1145Y; Abcam), MFN2 (6A8; Abcam), MULE (AX8D1; Cell Signaling Technology), β-TrCP (D13F10; Cell Signaling Technology), MID49 (16413-1-AP; Proteintech), BAK (B5897; Sigma), and BCLxL (2H12; BD Pharmingen). Alexa Flour 680-, IR800-, and HRP-conjugated secondary antibodies were purchased from Rockland and Southern Biotech. Detection was performed using an Odyssey Imaging System (Li-Cor) or enhanced chemiluminescence (Bio-Rad).

### Intracellular flow cytometry staining of MCL1

Cells were fixed in 1% paraformaldehyde (PFA) for 5 min, and then washed with FACS buffer (150 mM NaCl, 3.7 mM KCl, 2.5 mM CaCl_2_, 1.2 mM MgSO_4_, 1.2 mM KH_2_PO_4_, 0.8 mM K_2_HPO_4_ 14.8 mM HEPES, pH 7.2, 2% FCS). Fixed cells were incubated with rat monoclonal MCL1 antibody (19C4; WEHI antibody facility) for 30 min at 4 °C in FACS buffer containing 0.3% saponin followed by two washes with FACS buffer containing 0.03% saponin. Cells were then incubated with PE-conjugated anti-rat secondary antibody (Cat#3030-09; Southern Biotech) for 30 min at 4 °C in FACS buffer containing 0.3% saponin followed by two washes with FACS buffer containing 0.03% saponin. Cells were then analyzed using a flow cytometer (FACS Calibur; BD Biosciences).

### Immunofluorescence

Cells grown on coverslips were incubated with 250 nM of MitoTracker^TM^ Deep Red FM (Cat#,M22426; Thermo Fisher Scientific) for 30 min at 37 °C. Cells were subsequently fixed with 4% PFA for 15 min at 37 °C followed by three washes with PBS. Cells were permeabilized with 0.2% Triton X-100 in PBS containing 10% FCS for 20 min at room temperature, washed again with PBS, and then blocked with antibody dilution buffer (2.5% BSA in PBS) for 30 min. Permeabilized cells were incubated with rat monoclonal anti-MCL1 antibody (19C4; WEHI antibody facility) and mouse monoclonal anti-HA antibody (16B12; Covance) in antibody dilution buffer for 2 h with gentle shaking followed by three washes with PBS. Afterward, cells were incubated with AlexaFluor 555-conjugated goat anti-rat secondary antibody (Cat#A-21434; Thermo Fisher Scientific) and AlexaFluor 594-conjugated goat anti-mouse secondary antibody (Cat#11005; Thermo Fisher Scientific) for 1 h with gentle shaking followed again by three washes with PBS. Cells were also incubated with 0.5 µg/ml DAPI in PBS for 5 min before final three washes with PBS. Finally, cells were mounted onto a glass slide using SlowFade Diamond Antifade Mountant (Cat#S36963; Thermo Fisher Scientific) and coverslips were sealed with nail polish. Images were captured using a Zeiss LSM880 confocal microscope. At least ten fields of view were taken for each sample and images were analyzed using Fiji software.

### Cell viability assay

Cells were cultured with titrated concentrations of ABT-199 (Cat#A-1231, Active Biochem), A1331852 (Lessene Lab, WEHI), S63845 (Cat#A-6044, Active Biochem), MDM2 inhibitor RG7388 (Cat#C-1287, Chemgood), cisplatin (Sandoz), or etoposide (Sandoz) for 24–48 h. They were then suspended in KDS-BSS buffer containing 2 µg/ml propidium iodide. Cell viability was measured by assessing the exclusion of propidium iodide staining using a flow cytometer (FACS Calibur; BD Biosciences).

### Genome-wide CRISPR-Cas9 library screen

*Bax*^*−/*^^−^*Bak*^*−/−*^ MEFs were engineered through retroviral and lentiviral transduction to constitutively express GFP-MCL1, HA-tagged NOXA, and Cas9-T2A-mCherry. Cas9/mCherry^+ve^ GFP^low^ cells were sorted and expanded from single cells, and then transduced with a genome-wide lentiviral sgRNA library [24] at a multiplicity of infection of 0.3–1.3. Five to six days after transduction with the sgRNA library, GFP^high^ cells were sorted by flow cytometry and expanded. Flow sorting was repeated three times to further enrich cells containing sgRNAs that abrogated the ability of NOXA to degrade GFP-MCL1. Genomic DNA was extracted from the expanded cell populations using a DNeasy Blood and Tisuse Kit (Qiagen). sgRNA sequences were amplified by PCR using barcoded primers and quantitated by Illumina sequencing as previously described [23]. The screen was performed in duplicate using three independently derived cell clones, for a total of six biological replicate samples.

To identify genes whose sgRNAs were enriched in the sorted GFP^high^ cell populations, the sgRNA counts were first normalized to counts per million reads, then Log2 transformed and averaged across the six biological replicate samples for the postsort and presort cell populations. A Lowess curve was fitted to the relationship between postsort and presort values and residuals were calculated for each sgRNA. sgRNAs were ranked in descending order of their residual values, allowing minimum hypergeometric *p* values to be calculated for each gene in the library using an established algorithm [25]. These were subsequently corrected to false discovery rates using the Benjamini–Hochberg method.

### CRISPR-Cas9 gene targeting for generating knockout cell lines

Knockout cell lines were generated by delivering CRISPR-Cas9 gene targeting vectors into cells either by transient transfection or lentiviral transduction. Cas9 and sgRNA (Supplementary Table S1) were expressed using the following plasmids: pSpCas9(BB)-2A-GFP/PX458 (for transient co-expression of sgRNA and Cas9) [26], FUCas9Cherry (for constitutive lentiviral Cas9 expression) [23], pKLV-U6gRNA-EF(BbsI)-PGKpuro2ABFP (for constitutive lentiviral sgRNA expression) [24], and FgH1tUTG (for inducible lentiviral sgRNA expression) [23]. sgRNAs were expressed for at least 48 h before cells were sorted based on fluorescent marker protein expression. Individual clones were expanded from single sorted cells and successful knockout clones were identified by sequencing PCR amplicons encompassing the genomic loci targeted by the sgRNAs (Supplementary Table S2). In all cases where validated antibodies could be sourced, absence of the targeted proteins was also confirmed by western blotting.

### Cell growth competition assay

Cells expressing Cas9 were transduced with lentivirus encoding for the fluorescent marker protein BFP along with independent sgRNA targeting either *NOXA* or *MARCH5* (pKLV-U6gRNA-EF(BbsI)-PGKpuro2ABFP). Three days after infection, the cells were subjected to flow cytometry and an equal number of infected (BFP^+^^ve^) and uninfected (BFP^−ve^) cells were sorted and placed into the same well of a 12-well plate. The cells were cultured for 12 days and the proportion of BFP^+^^ve^ cells was measured every 3 days using a flow cytometer (LSRFortessa, BD Biosciences).

### *NOXA/PMAIP1* mRNA transcript analysis

*PMAIP1* mRNA expression data were sourced from the Cancer Cell Line Encyclopedia (http://www.broadinstitute.org/ccle), CellMiner (https://discover.nci.nih.gov/cellminer/), and the Multiple Myeloma Research Consortium (http://portals.broadinstitute.org/mmgp/). *Z*-scores of the mRNA expression were calculated and normalized based on *PMAIP1* mRNA from cell lines for which data appear on two or more databases.

### Statistical analyses and data replication

Formal statistical tests were applied only to ranking genes as hits to follow up from the sgRNA screen, the details of which are provided above and in the legend to Supplementary Table S3. The corresponding figure legends of all plotted data provide details with respect to how many independently derived knockout cell lines have been used, the number of times the experiments were replicated, the nature of the values plotted and their associated error bars. All immunoblot images shown are representative of data from at least two independent experiments.

## Results

### The degradation of MCL1 provoked by the BH3-only protein NOXA proceeds through a distinct pathway

To study how the BH3-only protein NOXA provokes MCL1 degradation, we first established a cellular system suitable for genetic and biochemical studies. Apoptosis relies on the essential mediators BAX and BAK and cannot proceed in their absence [27, 28]. By using *Bax*^−^^/−^*Bak*^−/−^ MEFs, we circumvented the downstream consequences of apoptosis signaling, such as the degradation of MCL1 by caspases [29].

**Fig. 1:**
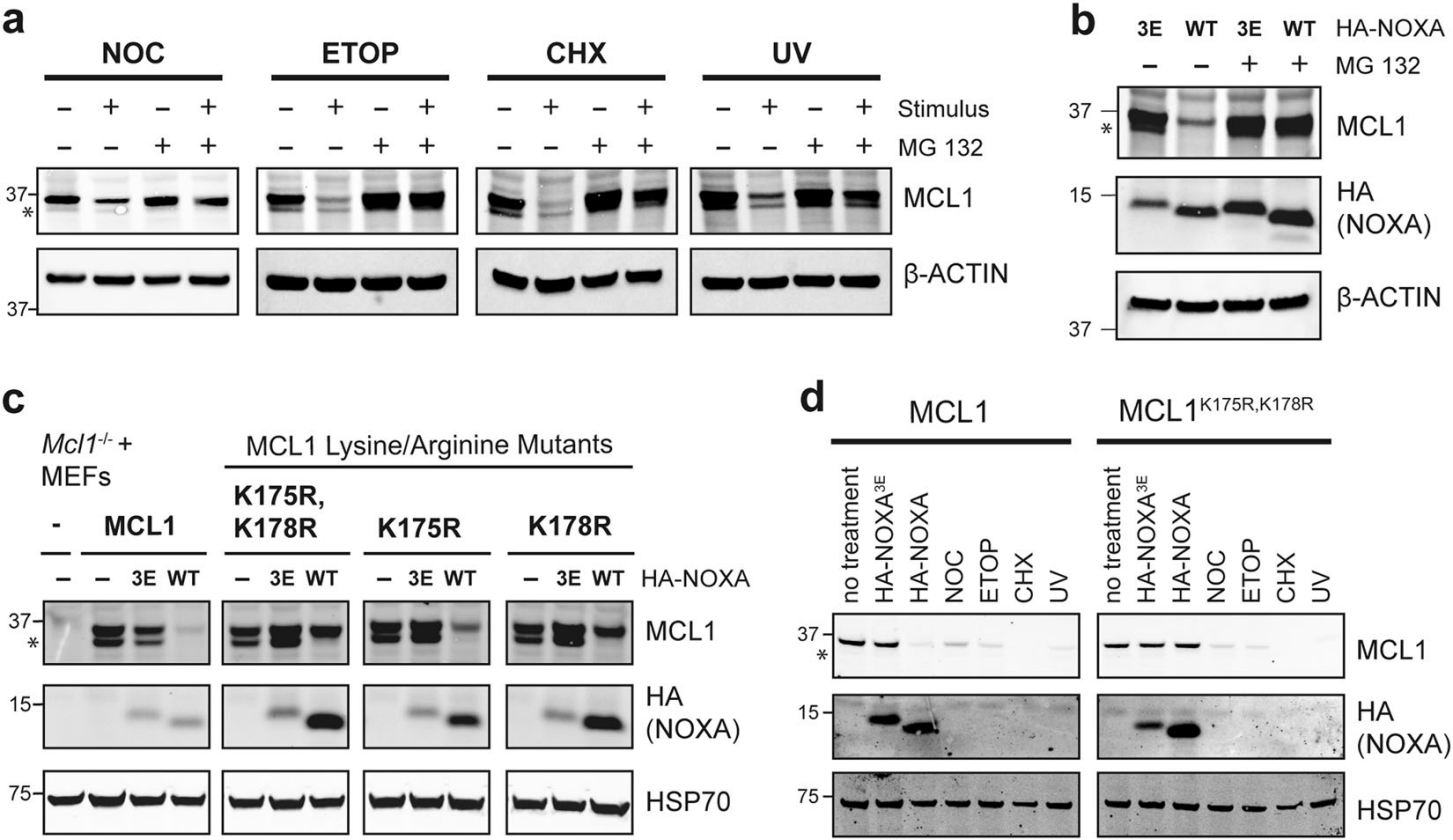
Lysines 175 and 178 of MCL1 are critical for its degradation triggered by the BH3-only protein NOXA, but not by other stimuli. **a** A variety of stress stimuli provoke MCL1 degradation by the proteasome. *Bax*^*−/−*^*Bak*^*−/−*^ MEFs were exposed to nocodazole (NOC; 400 ng/mL for 16 h), etoposide (ETOP; 50 μM for 16 h), cycloheximide (CHX; 50 μg/mL for 16 h), or UV irradiation (200 J/m^2^; then cultured for 4 h). Where indicated, the cells were also cultured with the proteasome inhibitor MG132 (10 μM). **b** NOXA promotes MCL1 degradation by the proteasome. *Bax*^*−/−*^*Bak*^*−/−*^ MEFs were engineered to express HA-NOXA (WT) or HA-NOXA^3E^ (3E), a mutant that does not bind MCL1 [21]. Where indicated, the cells were also cultured with the proteasome inhibitor MG132 (10 μM) for 16 h. **c** Lysines 175 and 178 of mouse MCL1 are critical for NOXA to trigger its degradation. *Mcl1*^*−/−*^*Bax*^*−/−*^*Bak*^*−/−*^ MEFs were engineered to express WT MCL1 or the indicated MCL1 mutants together with HA-NOXA or HA-NOXA^3E^. **d** Unlike NOXA-induced degradation, other stress stimuli degrade MCL1 independently of K175 and K178. *Mcl1*^*−/−*^*Bax*^*−/−*^*Bak*^*−/−*^ MEFs were engineered to express WT MCL1 or MCL1^K175R,K178R^ together with HA-NOXA or HA-NOXA^3E^, or exposed to nocodazole (NOC; 400 ng/mL for 16 h), etoposide (ETOP; 50 μM for 16 h), cycloheximide (CHX; 50 μg/mL for 16 h), or UV irradiation (200 J/m^2^; then cultured for 4 h). Asterisks denote MCL1^Matrix^, a form of the protein that has undergone N-terminal proteolysis associated with import into the mitochondrial matrix [67, 68]. The absence of MCL1^Matrix^ from cells expressing wild-type HA-NOXA suggests that the binding of NOXA to MCL1 may prevent such import from taking place.

Initially, we confirmed previous observations that a range of stress signals (growth arrest, DNA damage or protein synthesis inhibition) (Fig. 1a) and the enforced expression of NOXA (Fig. 1b) could all drive proteasomal degradation of MCL1 [9, 13, 15]. We noted that although NOXA caused MCL1 to be degraded by the proteasome, this was unperturbed in cells devoid of several E3 ligases known to ubiquitinate MCL1 in response to other signals, including MULE, β-TrCP, FBW7, and PARKIN (Supplementary Fig. S1) [10–13]. These observations suggest the distinct regulation of MCL1 by NOXA, and prompted us to ask whether certain lysines on MCL1 were required for NOXA-driven MCL1 degradation—this residue being the most common site for protein ubiquitination [30, 31].

To this end, we reconstituted *Mcl1*^−/−^*Bax*^−/−^*Bak*^−/−^ MEFs with WT mouse MCL1 or a series of mutants in which one or more of its 14 lysines were replaced with arginine. Ultimately, we focussed our attention on lysines 175 and 178 (Supplementary Fig. S2a). While replacing, these residues did not impact upon the ability of MCL1 to block apoptosis (Supplementary Fig. S2b) or to bind NOXA (Supplementary Fig. S2c), the MCL1^K175,K178R^ mutant was no longer targeted for degradation by NOXA (Fig. 1c). By contrast, its degradation in response to several other stress signals remained indistinguishable from WT MCL1 (Fig. 1d). Thus, lysine 175 and lysine 178 on MCL1 are uniquely required for NOXA-induced degradation.

Taken together, our data strongly suggest that a distinct molecular pathway is likely to drive the degradation of MCL1 when bound by NOXA, separate from those described for other signals, such as DNA damage-induced degradation of MCL1 by MULE [12].

### Genome-wide loss-of-function screen to identify genes required for MCL1 degradation by NOXA

To elucidate such a pathway, we undertook an unbiased CRISPR-Cas9 screen to identify genes required for NOXA to degrade MCL1. We introduced an N-terminally GFP-tagged form of MCL1 into *Bax*^−/−^*Bak*^−/−^ MEFs. While abundantly expressed, the GFP-tagged reporter was barely detectable when NOXA was co-expressed to provoke its degradation (Supplementary Fig. S3a). We anticipated that the level of the reporter would increase by genetically deleting factor(s) that were essential for NOXA to drive MCL1 turnover.

**Fig. 2:**
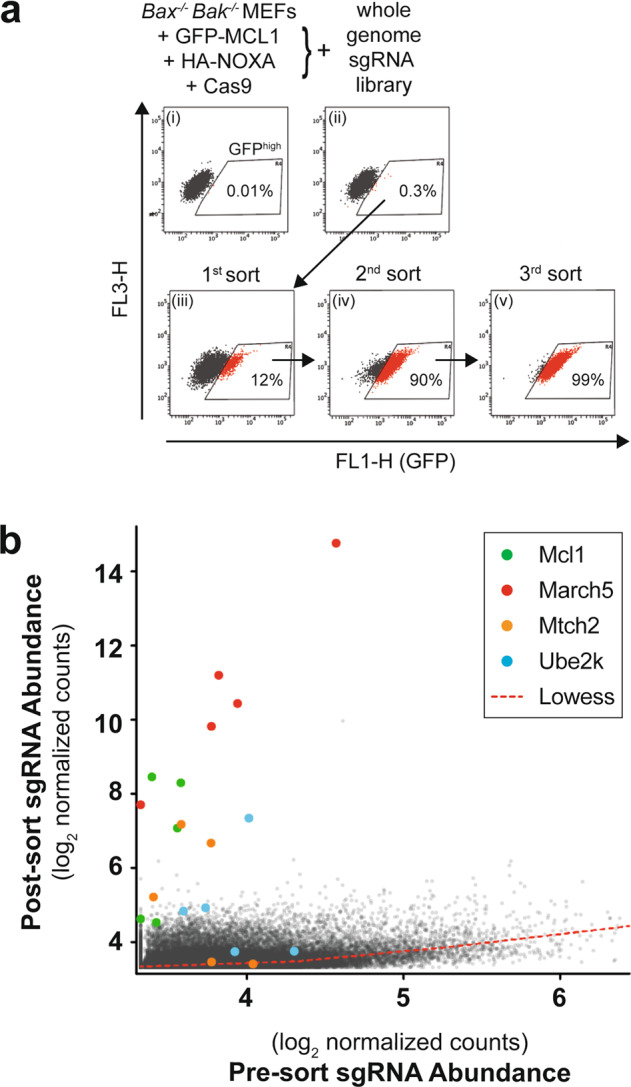
Genome-wide CRISPR-Cas9 screen for regulators of NOXA-induced MCL1 degradation. **a** Selection strategy to enrich cells in which the ability of NOXA to provoke MCL1 degradation has been disabled. Bax^−/−^Bak^−/−^ MEFs engineered to express GFP-MCL1 along with HA-NOXA and Cas9 (i) were transduced with a genome-wide sgRNA lentivirus library [24]. Following transduction, a small proportion of GFP^high^ cells was detected (ii) and enriched through three rounds of flow cytometric sorting (iii–v). **b** Quantitation of sgRNA abundance in the transduced cells before and after sorting. Multiple independent sgRNAs targeting *Mcl1, March5, Mtch2, and Ube2k* were enriched within the sorted GFP^high^ cell population. These data are also summarized in Supplementary Table S3.

A genome-wide lentiviral sgRNA library targeting mouse genes was introduced into independently derived MEF clones harboring the GFP-MCL1 reporter held in check by NOXA, upon which a small but distinct fraction of the cells became GFP^high^ (Fig. 2a and Supplementary Fig. S3b, c). These GFP^high^ cells were enriched to near homogeneity after multiple rounds of flow cytometric sorting (Fig. 2a). sgRNAs enriched by this selection process were identified by sequencing (Fig. 2b and Supplementary Table S3). One of the hits of the screen was *Mcl1* itself; while unexpected, we surmised that the *Mcl1* portion of the reporter gene would have been disrupted by *Mcl1* sgRNAs leaving behind the N-terminal GFP fragment linked to a truncated form of MCL1, now more stable and unable to bind NOXA.

Importantly, we also observed clear enrichment for multiple guides targeting three other genes: these encoded for the mitochondrial E3 ligase MARCH5, the E2 conjugating enzyme UBE2K and the mitochondrial outer membrane protein MTCH2 (Fig. 2b and Supplementary Table S3). Next, we sought to confirm a role for these genes in degrading MCL1.

### MARCH5, UBE2K, and MTCH2 are required for MCL1 degradation triggered by NOXA

**Fig. 3:**
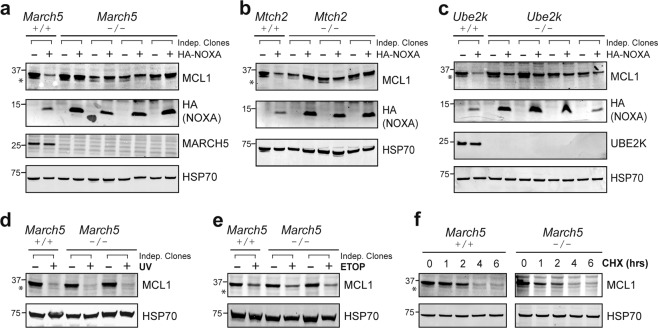
MARCH5, MTCH2, and UBE2K are critical for NOXA-induced MCL1 degradation. *March5* (**a**), *Mtch2* (**b**), or *Ube2k* (**c**) were deleted from *Bax*^*−/−*^*Bak*^*−/−*^ MEFs by CRISPR-Cas9 gene targeting. DNA sequencing was performed to confirm indel generation at sgRNA target sites (Supplementary Table S2). Confirmed knockout clones were then engineered to express HA-NOXA. The ability of NOXA to trigger MCL1 degradation was abrogated in the knockout cells. MARCH5 is not required for MCL1 degradation in MEFs subjected to DNA damage or protein synthesis inhibition. *Bax*^*−/−*^*Bak*^*−/−*^ and *Bax*^*−/−*^*Bak*^*−/−*^*March5*^*−/−*^ MEFs were exposed to **(d)** UV radiation (200 J/m^2^; then cultured for 4 h), **(e)** etoposide (50 μM for 16 h; ETOP), or **(f)** the protein synthesis inhibitor cycloheximide (50 μg/mL for up to 6 h; CHX). Asterisks denote MCL1^Matrix^, a truncated form of MCL1 that appears to be absent from cells expressing wild-type HA-NOXA for the reasons outlined in the legend to Fig. 1.

To validate the candidates identified by our screen, we assessed what impact their deletion had on the degradation of endogenous MCL1. Successful gene deletion was confirmed by DNA sequencing and, where antibodies were available, by the loss of protein expression (Fig. 3a–c). While NOXA promoted the degradation of MCL1 in the parental MEFs, deleting *March5* abrogated this in several independent clones (Fig. 3a). Interestingly, the levels of NOXA were concomitantly higher in cells lacking MARCH5, indicating that NOXA also became more stable when bound to MCL1 and the resulting protein complex was no longer marked for degradation. When bound to MCL1, NOXA was likely protected from additional ubiquitin-independent mechanisms that otherwise drive its rapid turnover [32]. Similar results were obtained with clones lacking MTCH2 (Fig. 3b) or UBE2K (Fig. 3c). Of note, the impact was less pronounced upon *Ube2K* deletion, suggesting that alternate E2 enzymes may supply ubiquitin to MARCH5 in the absence of UBE2K, as has been noted for E2s in other contexts [33, 34].

Despite the marked impact that deleting *March5, Mtch2*, or *Ube2k* had on MCL1 degradation triggered by NOXA overexpression in MEFs, little difference was observed on steady-state MCL1 levels in the knockout clones (Fig. 3a–c). Deleting *Noxa* also had little impact on MCL1 levels in MEFs (Supplementary Fig. S4a), suggesting that at steady-state MEFs express insufficient NOXA to meaningfully engage this pathway for degrading MCL1.

We explored further whether these genes contributed to MCL1 degradation triggered by other stress signals. However, consistent with our observation that NOXA provoked MCL1 degradation through a distinct pathway in MEFs (Fig. 1d), the absence of MARCH5, MTCH2, or UBE2K had little or no impact on MCL1 degradation caused by DNA damaging agents (Fig. 3d, e and Supplementary Fig. S4b, c) or on its basal turnover (Fig. 3f and Supplementary Fig. S4d). Thus, MARCH5, MTCH2, and UBE2K appear to operate in a specific pathway for degrading the MCL1:NOXA complex, which in MEFs becomes the most readily apparent when NOXA expression is elevated experimentally.

### NOXA provokes MCL1 degradation in diverse cells and influences steady-state MCL1 levels when abundantly expressed

**Fig. 4:**
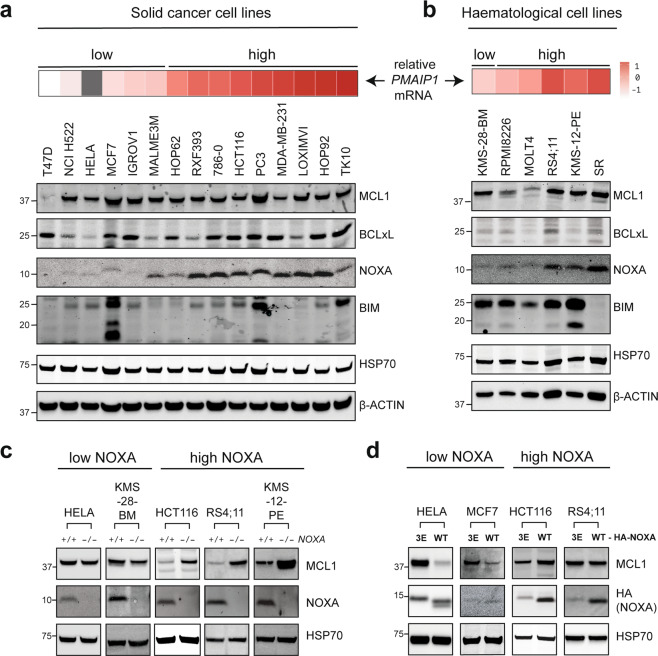
The steady-state level of MCL1 protein is controlled by NOXA when this BH3-only protein is abundantly expressed. Expression of select BCL2 family proteins in (**a**) solid and (**b**) hematological cancer cell lines. **c** In cells that express high levels of NOXA, this BH3-only protein controls steady-state MCL1 protein levels. *NOXA* was deleted using CRISPR-Cas9 gene targeting from a selection of cell lines that normally express either low (HELA, KMS-28-BM) or high (HCT116, RS4;11, KMS-12-PE) amounts of NOXA. Blots are representative of at least two independent knockout clones examined for each cell line. **d** HA-NOXA overexpression promoted MCL1 degradation in cell lines that normally express low amounts of NOXA (e.g., HELA and MCF7), but had minimal impact in those with high NOXA expression (e.g., HCT116 and RS4;11).

We postulated that some cells would express enough NOXA at steady-state to elicit constitutive MCL1 degradation by this pathway. To identify such examples, we examined NOXA protein expression across a large panel of cancer cell lines derived from both solid tumors (Fig. 4a) and hematological malignancies (Fig. 4b). There was a broad range of NOXA expression across these cell lines, allowing us to hone in on those with low NOXA expression (e.g., HELA, KMS-28-BM, and MCF7) or those with abundant NOXA (e.g., HCT116, RS4;11, and KMS-12-PE).

We predicted that modulating the *NOXA* expression would have different consequences in these two groups of cell lines, and this was indeed the case. In the cells that expressed minimal NOXA, MCL1 levels were largely unperturbed upon deleting *NOXA*, whereas MCL1 was degraded when exogenous NOXA was introduced (Fig. 4c, d), consistent with the responses we had observed in MEFs (Fig. 1b). Conversely, the cells with abundant NOXA had sufficient amounts to constitutively degrade MCL1. In these cells, deleting *NOXA* resulted in elevated steady-state MCL1 levels while overexpressing NOXA had little impact (Fig. 4c, d), presumably because the MARCH5 pathway was already fully engaged in these cells. Thus, NOXA can provoke MCL1 degradation in diverse cells and does so constitutively when expressed at sufficient levels.

### Context specific control of MCL1 by MARCH5 and MTCH2

**Fig. 5:**
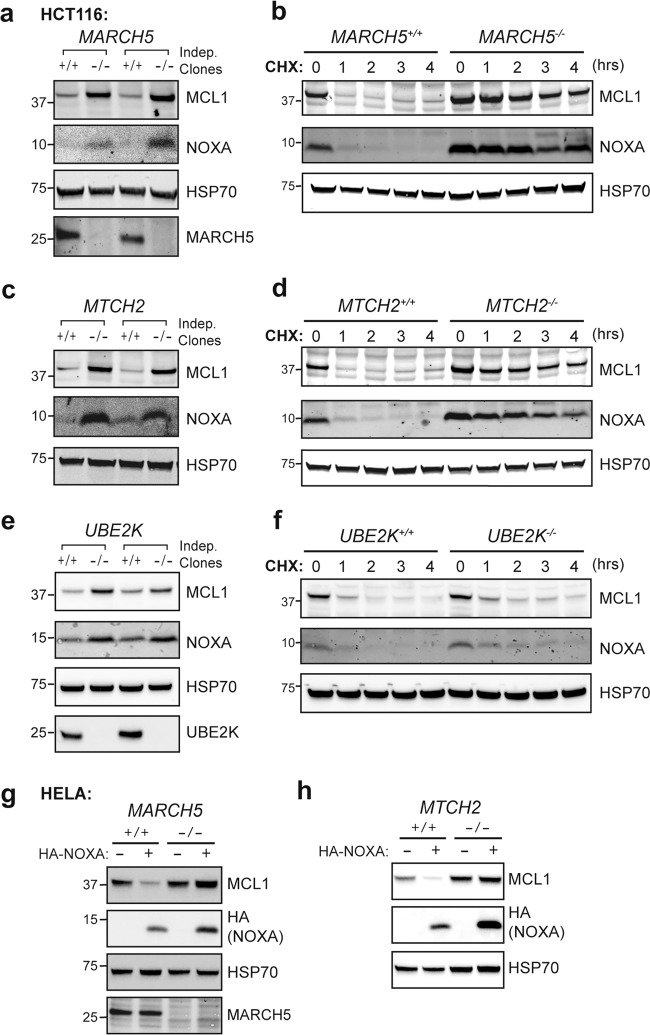
MARCH5 and MTCH2 control NOXA-driven MCL1 turnover in diverse cells. *MARCH5* (**a**, **b**), *MTCH2* (**c**, **d**) or *UBE2K* (**e**, **f**) were deleted from HCT116 cells by CRISPR-Cas9 gene targeting. Indel generation at sgRNA target sites was confirmed by DNA sequencing (Supplementary Table S2). Steady-state MCL1 expression (**a, c, e**) and the rate of MCL1 turnover (**b, d, f**) were examined in confirmed knockout clones. Cells were cultured with the protein synthesis inhibitor cycloheximide (50 μg/mL; CHX) for up to 4 h where indicated. MCL1 was markedly stabilized in clones lacking either MARCH5 or MTCH2 (**a**–**d**) and to a lesser degree in clones lacking UBE2K (**e**–**f**). *MARCH5* (**g**) or *MTCH2* (**h**) were deleted from HELA cells by CRISPR-Cas9 gene targeting. Indel generation at sgRNA target sites was again confirmed by DNA sequencing (Supplementary Table S2). MCL1 degradation caused by HA-NOXA overexpression was abolished in the knockout clones.

Given these results, we proceeded to explore whether the roles we had identified for MARCH5, MTCH2, and UBE2K in enabling NOXA to degrade MCL1 in MEFs were conserved in other cell types. We first tested whether deleting these genes would alter MCL1 levels in HCT116 cells, which express NOXA abundantly. Indeed, MCL1 was markedly stabilized in these cells upon deleting either *MARCH5* or *MTCH2* (Fig. 5a–d). Deleting *UBE2K* also stabilized MCL1 but only to a modest degree (Fig. 5e, f), which was consistent with the intermediate impact of *Ube2k* deletion observed in MEFs (Fig. 3c). The degradation of MCL1 in HELA cells upon enforced NOXA expression was also blocked by deleting either *MARCH5* or *MTCH2* (Fig. 5g, h).

Thus, the involvement of both MARCH5 and MTCH2 in NOXA-driven MCL1 degradation is conserved in diverse cells and can impact steady-state MCL1 levels in those that express high levels of NOXA. UBE2K, on the other hand, appears to be less critical. While it may serve as the principal E2 enzyme supplying ubiquitin to MARCH5 in some cases, alternative E2 partners can likely fulfill this role in the absence of UBE2K.

### Impaired turnover of the MCL1:NOXA complex does not enhance MCL1 prosurvival activity

Our results indicated that deleting *MARCH5* or *MTCH2* caused both NOXA and MCL1 to be stabilized concurrently (Figs. 3a–c and 5a–h). As stated above, this is likely because NOXA is rapidly degraded in a ubiquitin-independent manner when not bound to MCL1, and protected from such rapid turnover when engaged in complex [32]. Consistent with this hypothesis, NOXA was found to be extremely unstable in cells lacking MCL1 (Supplementary Fig. S5).

**Fig. 6:**
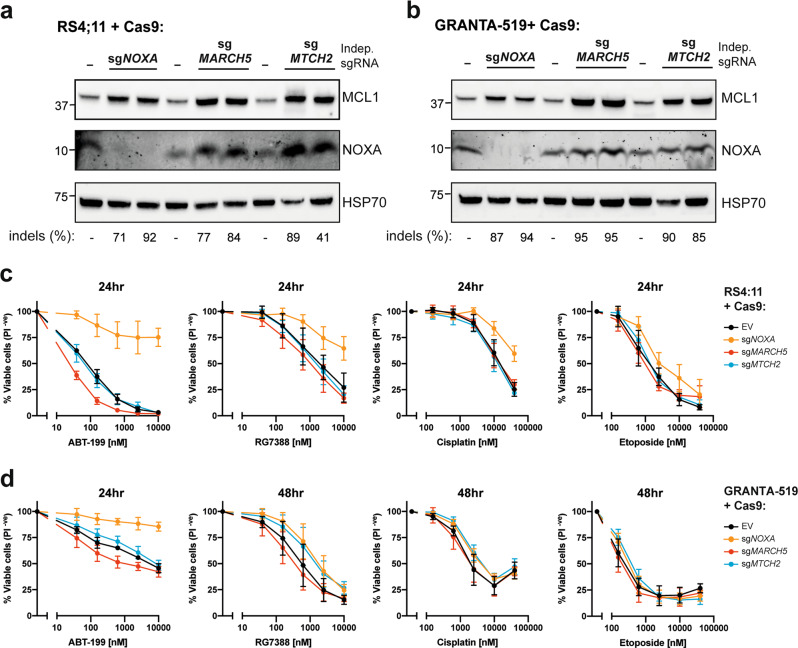
Distinct impacts on MCL1 prosurvival function upon deleting either *NOXA*, *MARCH5*, or *MTCH2*. Steady-state MCL1 protein levels were elevated in both RS4;11 (**a**) and GRANTA-519 (**b**) cells transduced with lentiviruses to express Cas9 and sgRNA targeting either *NOXA*, *MARCH5*, or *MTCH2*. The proportion of cells in the transduced populations bearing indels at the sgRNA target sites (as measured by DNA sequencing and reported in Supplementary Table S2) is indicated below the gels. Deleting *NOXA* rendered RS4;11 (**c**) and GRANTA-519 (**d**) cells less sensitive to several triggers of apoptosis, but deleting *MARCH5* and *MTCH2* did not despite MCL1 being stabilized to comparable levels in all cases. Transduced cell populations from **a** and **b** were exposed to the indicated concentrations of ABT-199, RG7388, cisplatin or etoposide for 24–48 h. Cell viability was measured by PI exclusion. Data represent mean ± standard deviation of three independent experiments using two independent sgRNA per gene.

The parallel changes in NOXA and MCL1 upon deleting *MARCH5* or *MTCH2* prompted us to consider the resulting impact on MCL1 prosurvival function. We evaluated this in the lymphoid cancer cell lines RS4;11 and GRANTA-519, both of which express high levels of NOXA and are sensitive to the BCL2 inhibitor ABT-199. MCL1 levels were stabilized in each of these cell lines following the deletion of either *NOXA*, *MARCH5*, or *MTCH2*, with NOXA levels rising in tandem with MCL1 upon removing *MARCH5* or *MTCH2* (Fig. 6a, b). Importantly, while deleting *NOXA* rendered both cell lines less sensitive to ABT-199 and several other apoptosis triggers, deleting *MARCH5* or *MTCH2* did not (Fig. 6c, d). Thus, the elevated levels of MCL1 that result from disabling turnover of the MCL1:NOXA complex do not give rise to elevated MCL1 prosurvival function, which is consistent with the notion that BH3-only proteins need not cause MCL1 degradation to provoke apoptosis [17].

Our findings indicate that deleting *MARCH5* has a very different outcome on the response of cells to apoptosis triggers compared with deleting *NOXA*. Notably, despite having elevated levels of MCL1 (Fig. 6a, b), RS4;11 and GRANTA-519 cells lacking MARCH5 were even more sensitive than their parental counterparts to some apoptosis triggers, particularly ABT-199 (Fig. 6c, d). We sought to confirm these results in two additional lymphoid cancer cell lines. Indeed, deleting *NOXA* also rendered both KMS-12-PE and RPMI8226 cells less sensitive BH3 mimetics (Supplementary Fig. S6a, b). However, these cell lines proved difficult to culture following transduction with lentiviruses to delete *MARCH5*. To quantitate our observation, we compared the relative outgrowth of gene-targeted cells in a competitive assay. Indeed, *MARCH5* gene-targeted cells exhibited a significant growth disadvantage in both KMS-12-PE and RPMI8226 cell lines, indicating that MARCH5 is essential for their fitness (Supplementary Fig. S6c–e). This observation is consistent with large-scale genetic screens that have found MARCH5 to be an essential gene in roughly one third of all human cancer cell lines [35].

Altogether, our data suggest that impairing MARCH5 E3 ligase function would not enhance MCL1 prosurvival activity despite stabilizing MCL1 protein levels. Modulating NOXA expression, on the other hand, should have a more significant impact in many cases as it influences both MCL1 stability and the ability of MCL1 to engage other BCL2 proteins. MARCH5 controls the turnover of multiple substrates in addition to MCL1, which may account for why we and others have found *MARCH5*^*−/−*^ cells to be more sensitive to certain triggers of apoptosis [36].

### MTCH2 cooperates with MARCH5 to degrade MCL1 but does not influence the turnover of another MARCH5 substrate: MID49

**Fig. 7:**
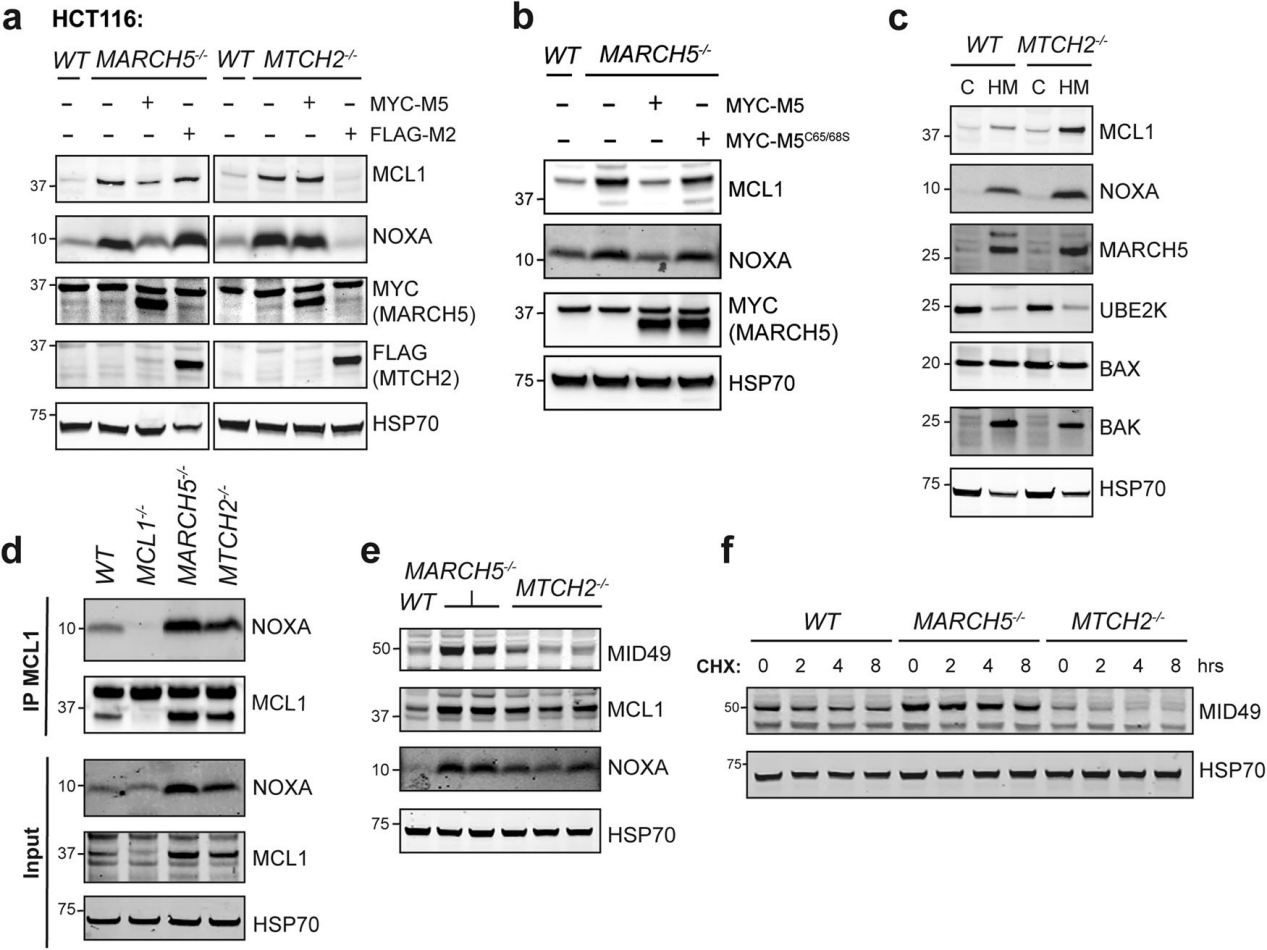
MTCH2 and MARCH5 jointly control turnover of the MCL1:NOXA complex. **a** MARCH5 and MTCH2 require each other to regulate MCL1 degradation by NOXA. *WT*, *MARCH5*^*−/−*^, or *MTCH2*^*−/−*^ HCT116 cells were engineered to stably express either MYC-MARCH5 (MYC-M5) or FLAG-MTCH2 (FLAG-M2). MYC-MARCH5 restored NOXA-driven MCL1 turnover in *MARCH5*^*−/−*^ but not *MTCH2*^*−/−*^ cells, while FLAG-MTCH2 did so in *MTCH2*^*−/−*^ but not *MARCH5*^*−/−*^ cells. **b** The E3 ubiquitin ligase function of MARCH5 is critical for regulating MCL1 degradation. *MARCH5*^*−/*^^−^ HCT116 cells were engineered to express either WT MARCH5 or a ligase-defective mutant (MARCH5^C65/68S^). While WT MARCH5 restored NOXA-driven MCL1 turnover, MARCH5^C65/68S^ did not. **c** MTCH2 is not required for the heavy membrane association of key proteins required for NOXA-driven MCL1 turnover. Protein lysates were prepared from cytosol (C) and heavy membrane (HM) fractions derived from *WT* and *MTCH2*^*−/−*^ HCT116 cells. **d** MCL1 was immunoprecipitated from HCT116 cells (*WT*, *MCL1*^*−/−*^, *MARCH5*^*−/−*^, and *MTCH2*^*−/−*^) cultured with proteasome inhibitor MG132 (10 μM for 8 h). NOXA continued to interact with MCL1 even when MTCH2 was absent. **e** The steady-state expression level of MID49 was elevated in *MARCH5*^*−/−*^ but not *MTCH2*^*−/−*^ HCT116 cells. **f** The rate MID49 protein turnover was reduced in *MARCH5*^*−/−*^ but not *MTCH2*^*−/−*^ HCT116 cells. Cells were cultured with the protein synthesis inhibitor cycloheximide (50 μg/mL; CHX) for up to 8 h.

The common requirement for both MARCH5 and MTCH2 to degrade MCL1 in diverse cells suggested that these proteins likely cooperate with each other in this process. To test this, we overexpressed FLAG-MTCH2 or MYC-MARCH5 in *MTCH2*^*−/−*^ and *MARCH5*^*−/−*^ HCT116 cells. The overexpressed proteins could compensate for their own absence to restore MCL1 degradation but MARCH5 overexpression could not overcome the absence of MTCH2 and likewise, MTCH2 overexpression could not overcome the absence of MARCH5 (Fig. 7a). Thus, MTCH2 and MARCH5 function in a mutually dependent manner to degrade the MCL1:NOXA complex.

MARCH5 possesses ubiquitin E3 ligase activity, enabling it to transfer ubiquitin to substrate proteins and mark them for degradation by the proteasome. We confirmed that the E3 ligase function of MARCH5 was required to drive MCL1 degradation, as reintroducing WT MARCH5 into *MARCH5*^−/−^ HCT116 cells restored MCL1 turnover, but reintroducing an E3 ligase-defective mutant (MARCH5^C65/68S^) did not (Fig. 7b).

In contrast to MARCH5, MTCH2 lacks enzymatic activity and it is not obvious how it might impact MCL1 stability. MTCH2 has been reported to interact with another BCL2 family protein, tBID, and regulate its localization to the mitochondria [37, 38]. We explored whether MTCH2 was also critical for tBID degradation, but it did not appear to be (Supplementary Fig. S7). We next tested whether MTCH2 controlled the subcellular localization of MARCH5, MCL1, or NOXA, but each of these proteins remained associated with mitochondria-enriched heavy membrane fractions in the absence of MTCH2 (Fig. 7c). NOXA and MCL1 also continued to interact with each other in the absence of MTCH2 (Fig. 7d). Thus, how MTCH2 cooperates with MARCH5 to degrade MCL1 remains unclear.

MARCH5 has been reported to target other mitochondrial proteins for degradation, of which MID49 is the best characterized [20, 36]. We therefore investigated whether MTCH2 also cooperated with MARCH5 to recognize and degrade this substrate. Although MID49 was stabilized in *MARCH5*^*−/−*^ HCT116 cells, its stability and rate of turnover were largely unaltered in cells lacking MTCH2 (Fig. 7e, f). This indicates that MTCH2 is not required for the degradation of all MARCH5 substrates and makes it unlikely that MTCH2 influences MCL1 degradation through a general impact on MARCH5 enzymatic activity. Instead, the impact of MTCH2 is more likely specific to either MCL1 or NOXA, perhaps influencing how the MCL1:NOXA complex is recognized by MARCH5.

### The C-terminal transmembrane domain of MCL1 is required for its degradation by MARCH5 and MTCH2

The ability of NOXA to provoke MCL1 degradation is unique among BH3-only proteins and is driven by specific amino acids within its BH3 domain [16]. Given the requirement for MARCH5 and MTCH2 to degrade the MCL1:NOXA complex, we next explored whether there were additional molecular requirements for MCL1 degradation to proceed. In particular, we noted that MARCH family E3 ligases often recognize their substrates through interactions between transmembrane domains [39–42]. We therefore postulated that the C-terminal transmembrane domain of MCL1 might contribute to its recognition and degradation by MARCH5.

**Fig. 8:**
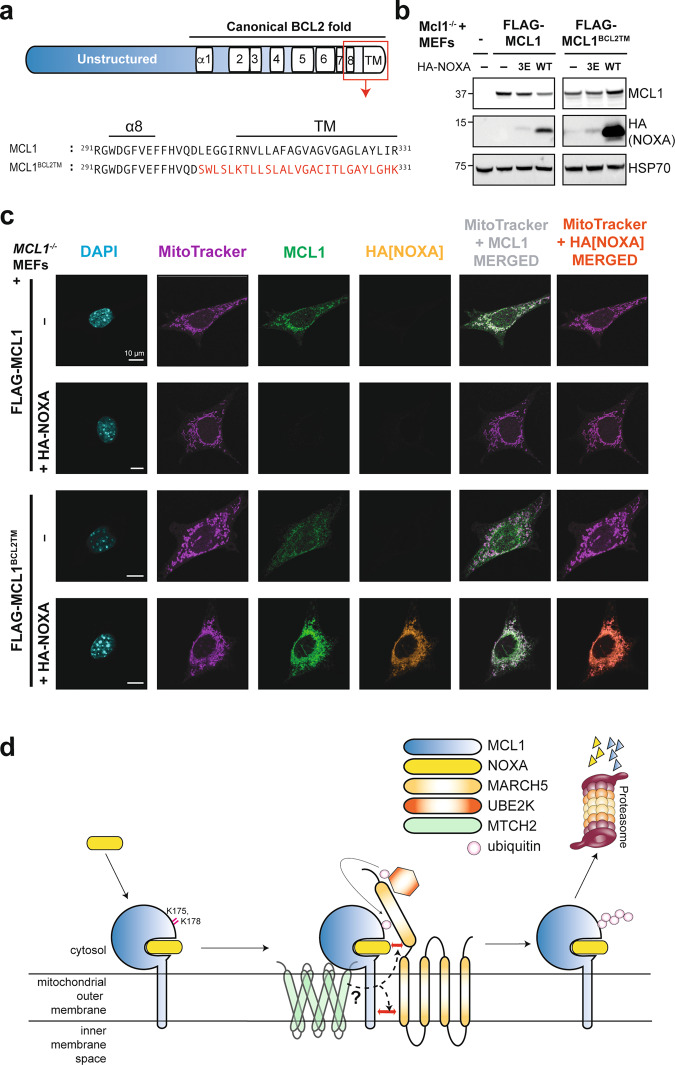
Elements of the MCL1 transmembrane domain are necessary for its turnover by MARCH5 and MTCH2. **a** Schematic highlighting the MCL1 transmembrane domain and a mutant in which this region of MCL1 was replaced by corresponding sequence from BCL2. **b** The MCL1 transmembrane domain is critical for NOXA to trigger MCL1 degradation. *Mcl1*^−/−^ MEFs were engineered to express FLAG-MCL1 or FLAG-MCL1^BCL2TM^ along with HA-NOXA or HA-NOXA^3E^. **c** *Mcl1*^−/−^ MEFs engineered to express FLAG-MCL1 or FLAG-MCL1^BCL2TM^ with or without HA-NOXA were imaged by confocal immunofluorescence microscopy. MCL1 (green) and NOXA (orange) were revealed by MCL1 and HA immunofluorescence, whilst DAPI (blue) and MitoTracker (purple) provided counterstain for the nucleus and mitochondrial network respectively. When co-expressed, FLAG-MCL1^BCL2TM^ and HA-NOXA were both present at high levels on the mitochondrial network (merged channels) indicating that degradation of the MCL1:NOXA complex by MARCH5/MTCH2 requires the MCL1 transmembrane domain. Scale bars: 10 μm. Images are representative of at least 10 fields of view captured for each condition. Equal laser intensities and thresholds were used in capturing and representing the MCL1 and HA immunofluorescence signals to faithfully represent their relative levels between samples. **d** Model highlighting key aspects of MARCH5-meditated turnover of MCL1. MARCH5 degrades MCL1 specifically when it is engaged by NOXA, suggesting that NOXA may contribute to the motif through which MARCH5 recognizes MCL1 as a substrate. Our findings also indicate that the integral mitochondrial outer membrane protein MTCH2, the transmembrane domain of MCL1, and key lysine residues on MCL1 are all critical elements of this mechanism.

To test this idea directly, we replaced the membrane targeting region of MCL1 with equivalent residues from a related prosurvival protein, BCL2 (MCL1^BCL2TM^; Fig. 8a). These substitutions did not affect the ability of MCL1 to block apoptosis (Supplementary Fig. S8a) or to bind NOXA (Supplementary Fig. S8b), but did prevent NOXA from causing its degradation (Fig. 8b). Subcellular fractionation and confocal imaging confirmed that MCL1^BCL2TM^ was properly localized at heavy membranes including mitochondria, nuclear envelope and endoplasmic reticulum (Fig. 8c and Supplementary Fig. S8c), consistent with the intracellular distributions of both BCL2 and MCL1 [43, 44]. Despite being distributed slightly more broadly than MCL1 itself, the MCL1^BCL2TM^ localized at mitochondria was clearly not degraded when co-expressed with NOXA (Fig. 8c). Hence, we conclude that in addition to motifs contributed by the BH3 domain of NOXA within the MCL1:NOXA complex [16], elements of the MCL1 transmembrane domain are also necessary for recognition by MARCH5. Along with the requirement for MTCH2 in this process and our identification of specifically required lysine residues on MCL1, we can begin to formulate a model for how these observations fit together (Fig. 8d). We postulate that MARCH5 and MCL1 may interact directly, potentially through their transmembrane domains, and possibly aided by a second interface involving NOXA. MTCH2 may also bridge interactions that take place within or adjacent to the mitochondrial outer membrane. We propose that these proteins come together in a specific orientation that allows MARCH5 to discharge ubiquitin onto K175/K178 of MCL1, thereby marking it for degradation.

## Discussion

It has been recognized for some time that that the MCL1:NOXA complex is rapidly degraded by the proteasome, but the mechanism responsible has remained unclear [15, 16]. Using a genome-wide CRISPR-Cas9 screen we have now identified that MARCH5 and MTCH2 are both required for this process and elucidated certain aspects of how they work together.

We had previously identified a motif within the NOXA BH3 domain that is important for causing MCL1 degradation [16]. MARCH5 may recognize the MCL1:NOXA complex in part through this motif (Fig. 8d). We have now identified that the transmembrane domain of MCL1 also appears to be important, which is consistent with the notion that E3 ligases of the MARCH family commonly recognize their integral membrane protein substrates through interactions within the lipid bilayer [39–42].

Our data indicate that two MCL1 lysine residues (K175 and K178) are necessary for degradation of the MCL1:NOXA complex. MARCH5 likely transfers ubiquitin to one or both of these sites, thereby marking MCL1 for degradation by the proteasome. The nature of the ubiquitin chain and whether it is formed on one or both of these lysine residues remains to be determined. Our data do however indicate that the degradation of MCL1 is partly impaired when either of K175 or K178 is removed, and completely abrogated when both are absent (Fig. 1c). The strict requirement for specific lysine residues is notable. In most cases E3 ligases can discharge ubiquitin onto a wide range of nearby accessible lysine residues. The MCL1 E3 ligase MULE, for example, could drive MCL1 degradation by ubiquitinating any of several lysine residues within its unstructured N-terminus [12]. That MARCH5 specifically requires K175 and K178 may imply that MCL1, NOXA, and MARCH5 must come together in a precise orientation to produce the correct environment for ubiquitin discharge to occur.

We show that MARCH5 and MTCH2 cooperate to degrade the MCL1:NOXA complex in a variety of cell types, indicating that this mechanism is well conserved. Whilst our study does not specifically address where this degradation pathway has the greatest biological influence, we postulate that it may be most important in contexts where the ability of NOXA to counteract MCL1 is critical. Possibilities include the development of B- and T-cells and their responses to antigen [45, 46] as well as apoptotic responses to viral infection [47, 48]. Notably, the induction of NOXA during viral infection is controlled in part by MAVS [49]—a key regulator of antiviral signaling and another substrate of MARCH5 [50]. It is also becoming increasingly clear that NOXA can significantly influence the response of cancer cells to drug therapies, particularly BH3 mimetics such as venetoclax, as NOXA loss has been found to underpin resistance to these agents in lymphoid malignancies [51–53]. Our findings confirm that NOXA can indeed be critical for the response of cancer cells to BH3 mimetics (Fig. 6c, d and Supplementary Fig. S6a, b).

The influence of MTCH2, an integral mitochondrial outer membrane protein, on NOXA-driven MCL1 degradation is also particularly intriguing. MTCH2 has not previously been linked to protein degradation, but has been reported to influence BCL2 family proteins through facilitating recruitment of the BH3-only protein tBID to mitochondria and thereby promoting apoptosis induction [37, 38, 54]. However, in relation to MCL1 degradation by MARCH5, MTCH2 deletion did not appear to significantly impact the localization of MCL1, NOXA, or MARCH5 (Fig. 7c). It is possible that MTCH2 serves as an adaptor protein that bridges interactions between MARCH5 and the MCL1:NOXA complex within the mitochondrial outer membrane (Fig. 8d).

Although the precise function of MTCH2 at mitochondria remains uncertain, prior reports have primarily linked MTCH2 to two biological roles: cellular metabolism and apoptosis. The link to metabolism originates from the identification of MTCH2 as a genetic susceptibility locus for obesity [55, 56], with subsequent studies in conditional knockout mice demonstrating altered metabolic profiles in cells lacking MTCH2 [57, 58]. MTCH2 has also been associated with regulating mitochondrial fission and fusion dynamics [59]. The association with apoptosis, on the other hand, has until now centered on the ability of MTCH2 to recruit tBid to mitochondria [37, 54]. Our findings now associate MTCH2 with regulating additional BCL2 family proteins: MCL1 and NOXA. These findings strengthen the connection between MTCH2 and BCL2-regulated apoptosis and raise the possibility that MTCH2 may have a broader role in linking metabolism to apoptosis regulation.

The mechanism through which NOXA, MTCH2, and MARCH5 cooperate to drive MCL1 turnover may help to inform novel strategies for promoting MCL1 degradation as an alternative to inhibiting MCL1 with selective BH3 mimetics. MCL1 itself remains a highly compelling drug target. Although MCL1-selective inhibitors have been developed [60–62], the critical importance of MCL1 in diverse healthy tissues suggests that the therapeutic window associated with inhibiting MCL1 systemically may be narrow. We anticipate that this will be especially true in contexts where there would be advantages to combining MCL1 inhibitors with complimentary BCL2 protein inhibitors such as venetoclax. In such scenarios, the ability to inhibit MCL1 in a tissue or disease specific manner rather than systemically would be highly valuable. Harnessing protein degradation pathways, such as with PROTAC small molecules, is one way to achieve such contextual inhibition [63], and this is indeed a strategy being pursued for MCL1 [64, 65]. Distinct from PROTACs, compounds that act as “molecular glues” to enhance the activity of a cognate E3 ligase on its substrate have also recently been described [66]. The novel details that we have elucidated for NOXA-driven MCL1 degradation could be highly informative for strategies such as these.

## Supplementary information

Supplementary Figure 1

Supplementary Figure 2

Supplementary Figure 3

Supplementary Figure 4

Supplementary Figure 5

Supplementary Figure 6

Supplementary Figure 7

Supplementary Figure 8

Supplementary Table 1

Supplementary Table 2

Supplementary Table 3

Supplementary Figure and Table Legends
