# Supplementary figures and images for: MARCH5 requires MTCH2 to coordinate proteasomal turnover of the MCL1:NOXA complex
Source: Cell Death Differ. 2020 Feb 24;27(8):2484–99. doi: 10.1038/s41418-020-0517-0 (PMC7370232; doi:10.1038/s41418-020-0517-0)

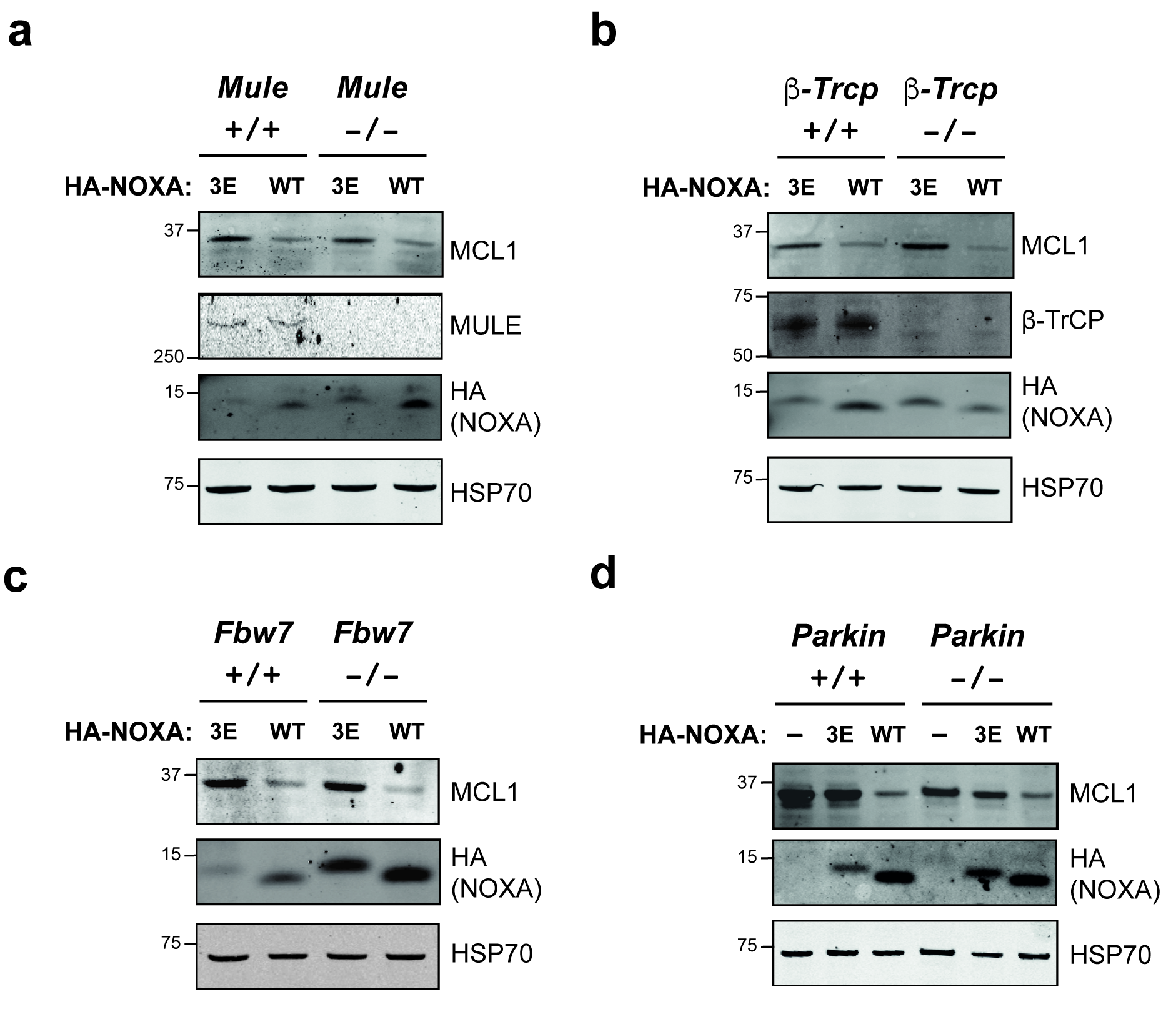

Supplement: Supplementary file 1 — Supplementary Figure 1 [file 41418_2020_517_MOESM1_ESM.tif]

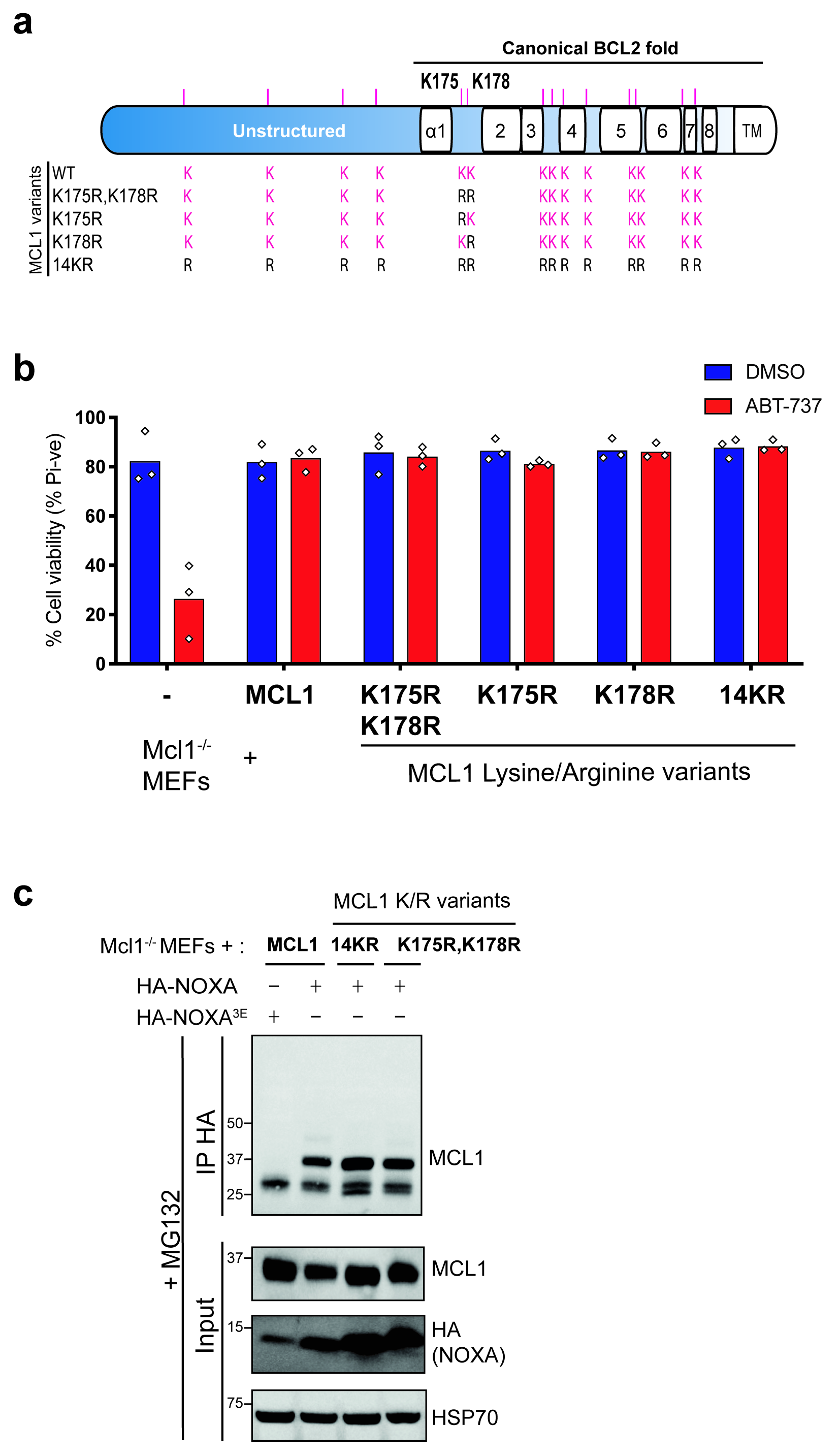

Supplement: Supplementary file 2 — Supplementary Figure 2 [file 41418_2020_517_MOESM2_ESM.tif]

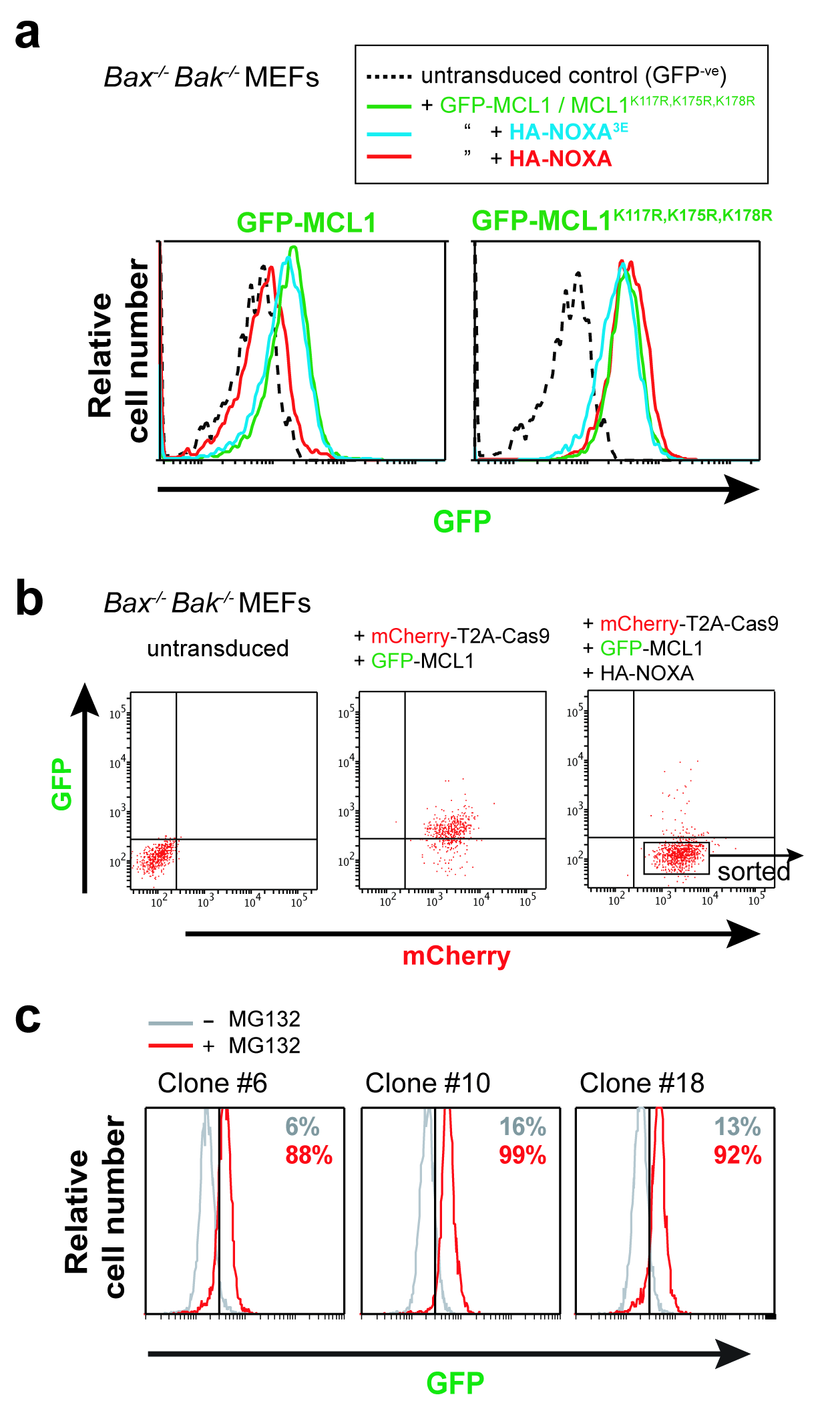

Supplement: Supplementary file 3 — Supplementary Figure 3 [file 41418_2020_517_MOESM3_ESM.tif]

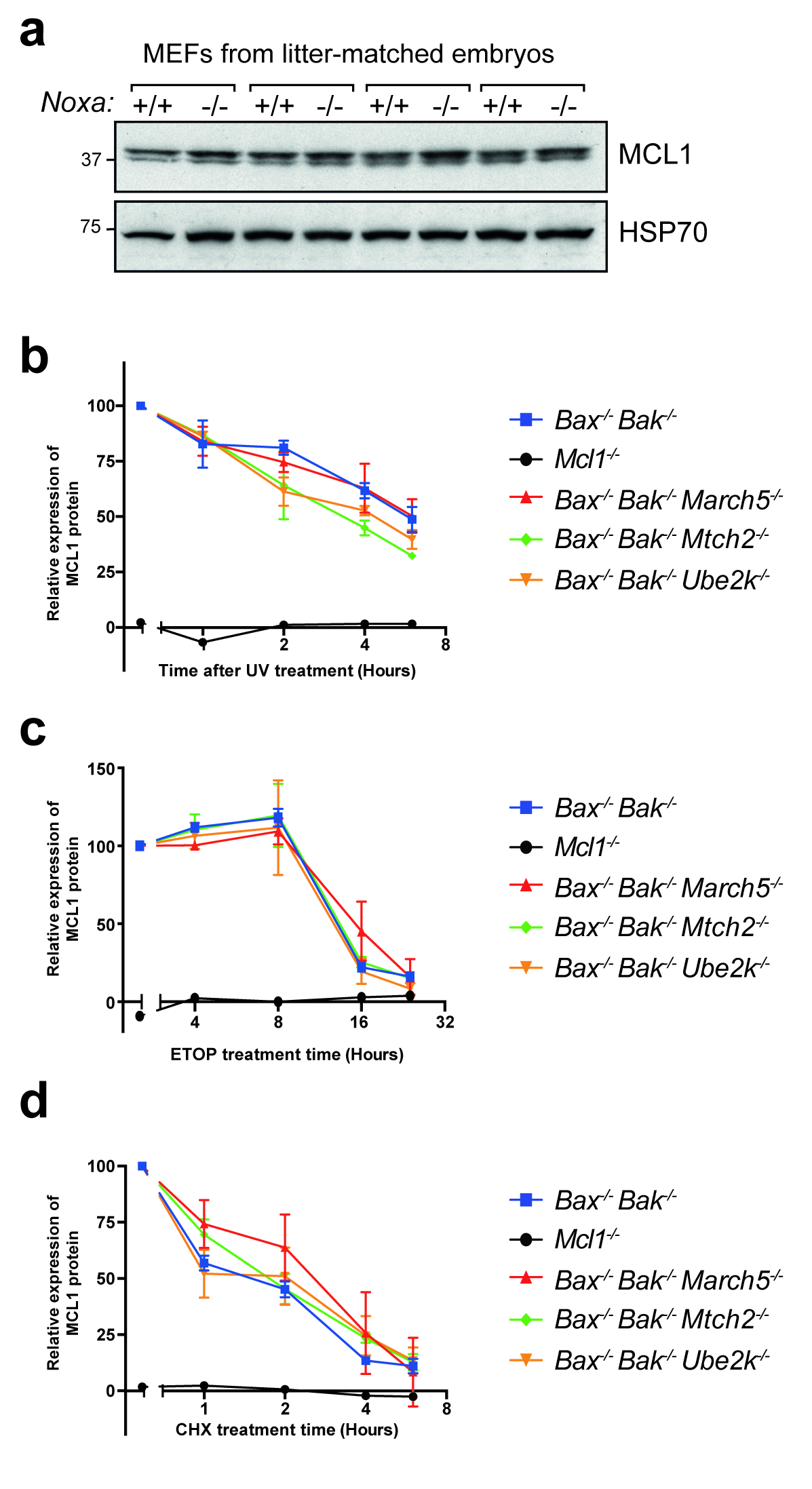

Supplement: Supplementary file 4 — Supplementary Figure 4 [file 41418_2020_517_MOESM4_ESM.tif]

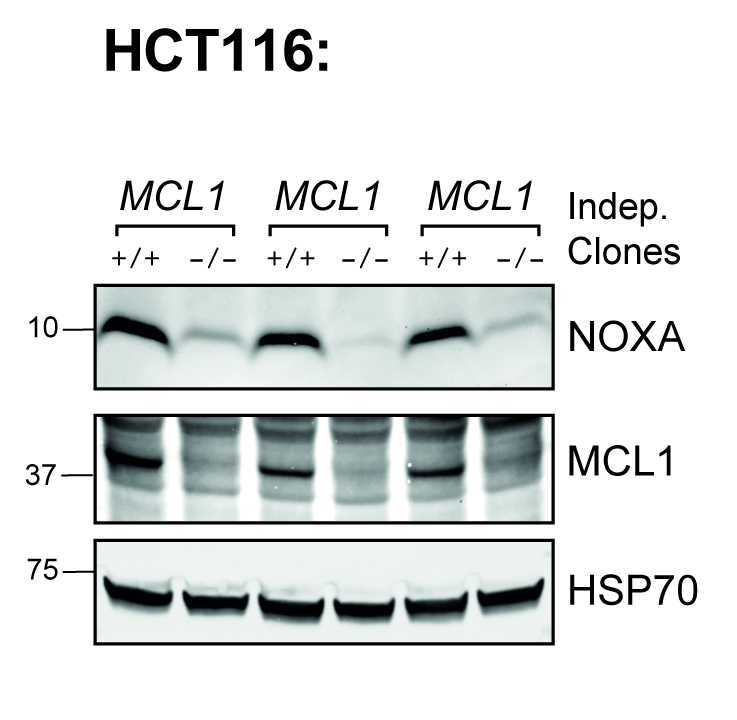

Supplement: Supplementary file 5 — Supplementary Figure 5 [file 41418_2020_517_MOESM5_ESM.tif]

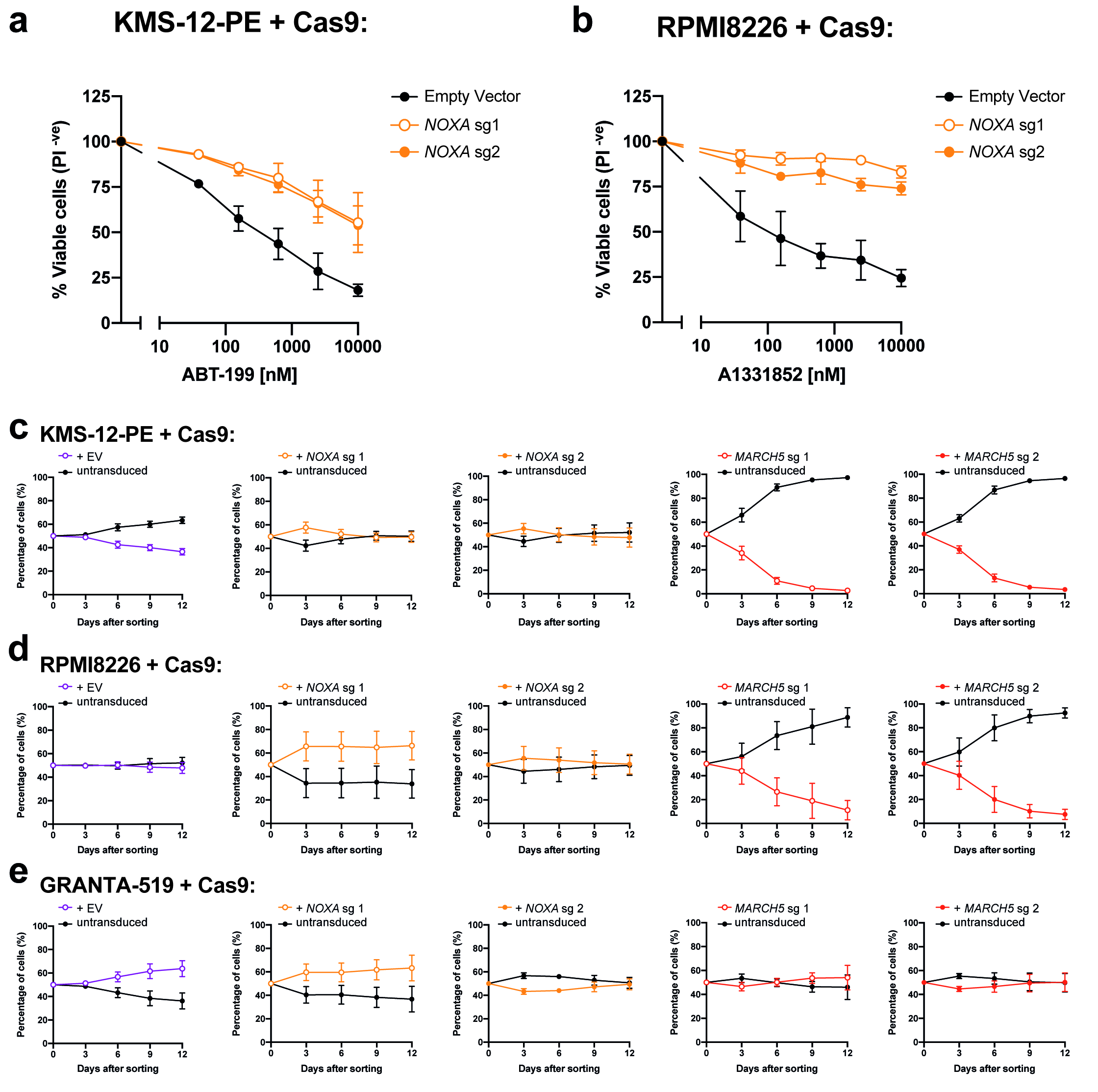

Supplement: Supplementary file 6 — Supplementary Figure 6 [file 41418_2020_517_MOESM6_ESM.tif]

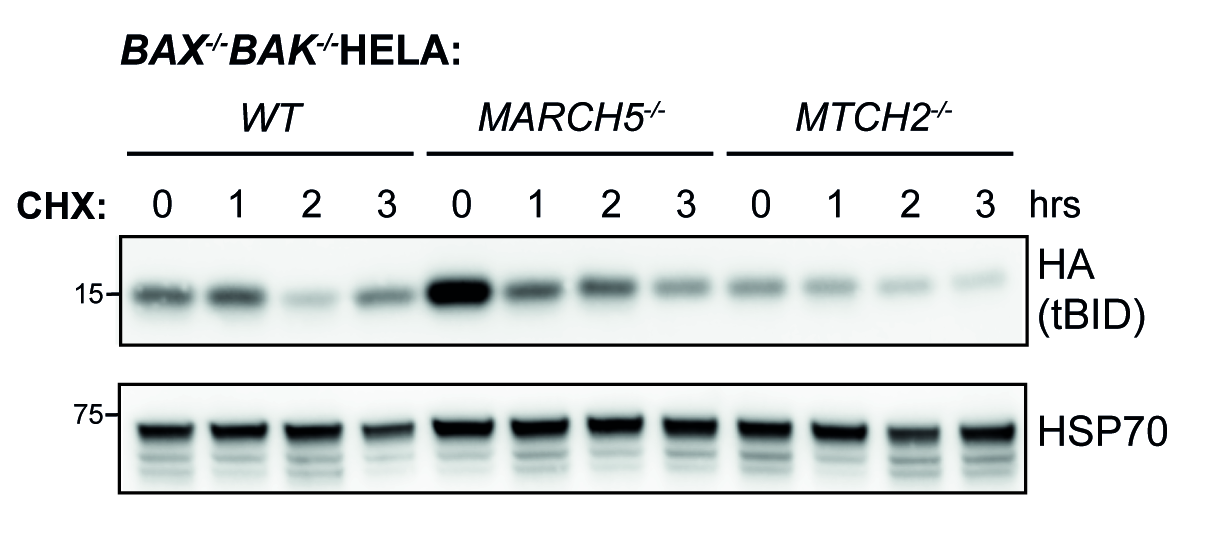

Supplement: Supplementary file 7 — Supplementary Figure 7 [file 41418_2020_517_MOESM7_ESM.tif]

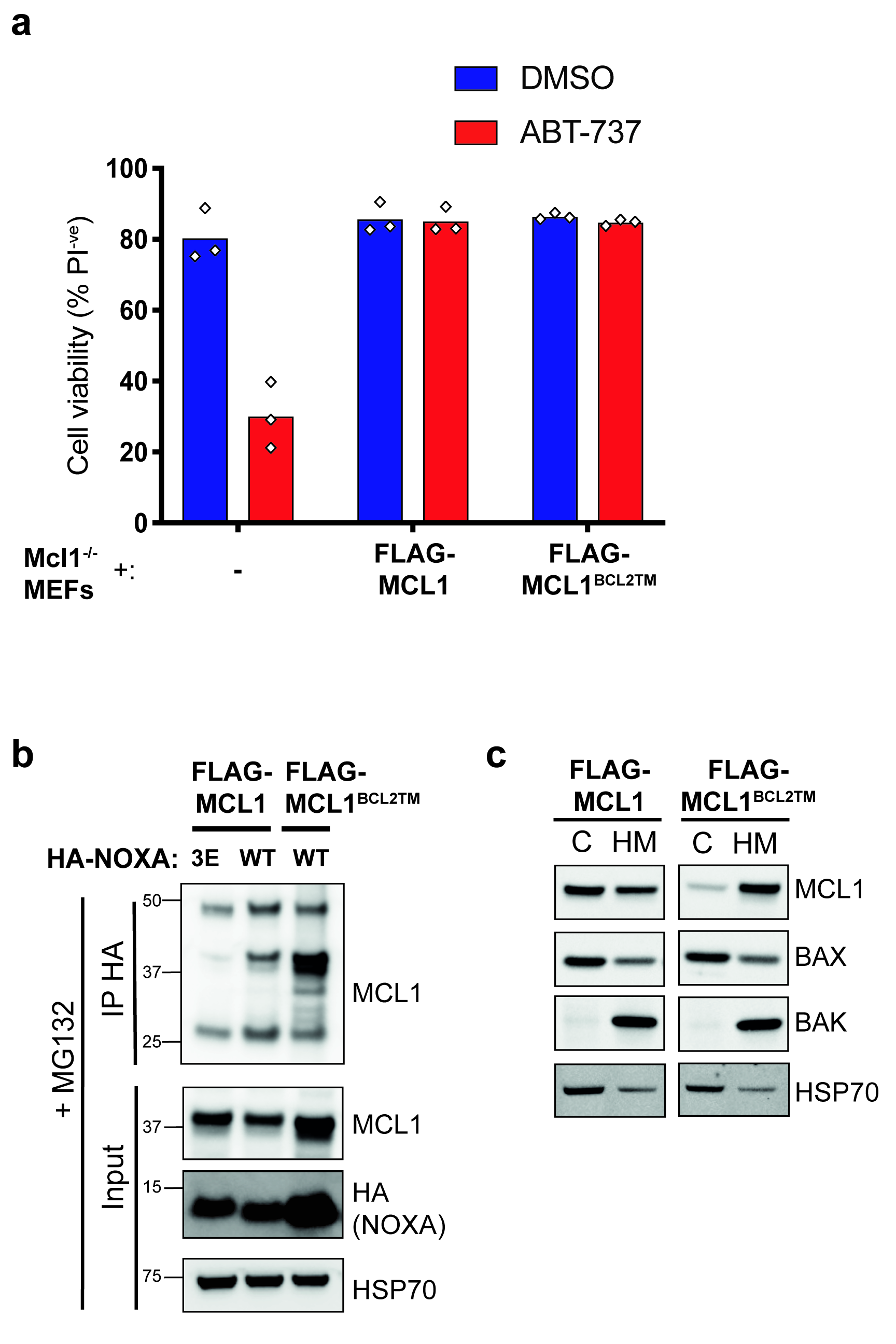

Supplement: Supplementary file 8 — Supplementary Figure 8 [file 41418_2020_517_MOESM8_ESM.tif]
