# Supplementary material for: MARCH5 requires MTCH2 to coordinate proteasomal turnover of the MCL1:NOXA complex
Source: Cell Death Differ. 2020 Feb 24;27(8):2484–99. doi: 10.1038/s41418-020-0517-0 (PMC7370232; doi:10.1038/s41418-020-0517-0)
Supplement: Supplementary file 12 — Supplementary Figure and Table Legends [file 41418_2020_517_MOESM12_ESM.docx]

**Supplementary Figure S1**

**The ubiquitin E3 ligases MULE, β-TRCP, FBW7 and PARKIN are not required for NOXA-mediated MCL1 degradation.**

**(a)** *Mule,* **(b)** *β-Trcp,* **(c)** *Fbw7* and **(d)** *Parkin* were deleted from wild-type **(a)** or *Bax^-/-^Bak^-/-^* MEFs **(b-d)** by CRISPR-Cas9 gene targeting. Indel generation at sgRNA target sites was confirmed by DNA sequencing (Supplementary Table S2). Confirmed knockout clones were then engineered to express HA-NOXA or HA-NOXA^3E^. Blots are representative of at least two independent knockout clones examined for each gene.

**Supplementary Figure S2**

**MCL1 lysine/arginine replacement variants retain their pro-survival function and their ability to bind NOXA.**

1. Schematic indicating lysine/arginine replacement mutations made to MCL1.
2. All MCL1 lysine/arginine replacement mutants retained their ability to inhibit apoptosis. *Mcl1^-/-^* MEFs were engineered to express WT MCL1, MCL1^K175R,K178R^, MCL1^K175R^, MCL1^K178R^ or MCL1^14KR^ then exposed to ABT-737 (2 μM) for 24 h. Cell viability was measured by propidium iodide exclusion. n = 3 independent experiments.
3. All MCL1 lysine/arginine replacement mutants retained their ability to bind NOXA. *Mcl1^-/-^* MEFs were engineered to express WT MCL1, MCL1^14KR^, or MCL1^K175R,K178R^ together with HA-NOXA or HA-NOXA^3E^, a mutant that does not bind MCL1. HA immunopreciptiations were performed on lysates prepared from cells cultured with the proteasome inhibitor MG132 (10 μM) for 8 h.

**Supplementary Figure S3**

**Characterization of GFP-MCL1 reporter and clones utilized for CRISPR-Cas9 screen.**

1. GFP-tagged MCL1 is targeted for degradation upon co-expressing NOXA. *Bax^-/-^Bak^-/-^* MEFs were engineered to express GFP-MCL1 or GFP-MCL1^K117R,K175R,K178R^ and also HA-NOXA or HA-NOXA^3E^ where indicated.
2. Selection of clones for CRISPR-Cas9 screen. *Bax^-/-^Bak^-/-^* MEFs were engineered to express GFP-MCL1 together with Cas9-T2A-mCherry and HA-NOXA. Single cells with low GFP-MCL1 expression (resulting from HA-NOXA co-expression) were sorted and expanded from the population.
3. Characterization of clones utilized for screen. Individual clones were sorted and expanded (as described in **b**) and then exposed to proteasome inhibitor MG132 (10 μM) for 6 h. Each of the selected clones exhibited robust recovery of GFP-MCL1 expression following MG132 treatment.

**Supplementary Figure S4**

**The NOXA-dependent pathway for degrading MCL1 involving MARCH5, MTCH2 and UBE2K is not required for MCL1 degradation caused by DNA damage or protein synthesis inhibition in MEFs.**

1. Deleting *Noxa* has no discernible impact on MCL1 stability in MEFs.

**(b-d)** MARCH5, MTCH2 and UBE2K are dispensable for MCL1 degradation induced by exposure to UV radiation, etoposide, or cycloheximide. *Bax*^-/-^ *Bak*^-/-^ MEFs and clones in which *March5*, *Mtch2*, or *Ube2k* were deleted by CRISPR-Cas9 gene targeting were exposed to **(b)** 200 J/m^2^ UV radiation, **(c)** 50 μM etoposide (ETOP), or **(d)** 50 μg/mL cycloheximide (CHX). Cells were harvested at the indicated times and MCL1 protein levels were determined by intracellular FACS staining. *Mcl1*^-/-^ MEFs served as a negative control. Data presented as mean ± standard error of mean of biological replicates using independently derived knockout clones (n=3 for *March5^-/-^ Bax*^-/-^ *Bak*^-/-^, *Ube2k^-/-^* *Bax*^-/-^ *Bak*^-/-^ or *Mtch2^-/-^* *Bax*^-/-^ *Bak*^-/-^ MEFs) and (n=2 for *Bax*^-/-^ *Bak*^-/-^ (control) and UV-treated *Mtch2^-/-^* *Bax*^-/-^ *Bak*^-/-^ MEFs).

**Supplementary Figure S5**

**NOXA is highly unstable in the absence of MCL1**.

*MCL1^-/-^* HCT116 clones were generated by CRISPR-Cas9 gene editing. *MCL1* gene disruption was verified in the knockout clones by DNA sequencing (Supplementary Table S2). NOXA protein levels were dramatically reduced in the absence of MCL1.

**Supplementary Figure S6**

**Distinct impacts of deleting *NOXA* and *MARCH5*** **in lymphoid cancer cell lines.**

**(a-b)** Deleting *NOXA* rendered KMS-12-PE and RPMI8226 cells resistant to selective BH3 mimetics. KMS-12-PE **(a)** and RPMI8226 **(b)** cells transduced with lentiviruses to express Cas9 and independent sgRNA targeting *NOXA*. Indel generation at the *NOXA* locus was confirmed to be ≥ 90% in all cases (Supplementary Table S2). Gene-edited cell popoulations were then exposed to titrated concentrations of the BCL-2 inhibitor ABT-199 **(a)** or the BCL-XL inhibitor A1331852 **(b)** for 24 h. Cell viability was measured by PI exclusion. Data represent mean ± standard deviation of three independent experiments.

**(c-e)** Cell growth competition assay. KMS-12-PE **(c)**, RPMI8226 **(d)**, and GRANTA-519 **(e)** cells expressing Cas9 were tranduced with a control lentivirus (Empty Vector) encoding only for the fluorescent marker protein BFP or with lentiviruses encoding for both BFP and independent sgRNA targeting either *NOXA* or *MARCH5*. Equal numbers of transduced cells (BFP^+ve^) and untransduced cells (BFP^-ve^) were sorted from the cultures 72 hours after infection. Sorted cells were co-cultured for 12 days, with the proportion of BFP^+ve^ cells measured every three days. Whilst GRANTA-519 cells could tolerate MARCH5 deletion **(e)**, both KMS-12-PE and RPMI8226 cells exhibited a marked loss of fitness upon MARCH5 deletion (**c-d**). Data represent mean ± standard deviation of three independent experiments.

**Supplementary Figure S7**

**MTCH2 and MARCH5 do not play major roles in turnover of the BH3-only protein tBID.**

*BAX^-/-^BAK^-/-^* HELA cells and clones lacking either MARCH5 or MTCH2 were engineered to express HA-tBID. Whole cell protein extracts were prepared from cells cultured with the protein synthesis inhibitor cycloheximide (50 μg/ml; CHX) for up to 3 h.

**Supplementary Figure S8**

**MCL1^BCL2TM^ remains associated with heavy membranes, retaining its pro-survival function and ability to bind NOXA.**

1. MCL1^BCL2TM^ retains the ability to inhibit apoptosis. *Mcl1*^-/-^ MEFs were engineered to express FLAG-MCL1 or FLAG-MCL1^BCL2TM^ then exposed to ABT-737 (2 μM) for 24 h. Cell viability was measured by propidum iodide exclusion. n = 3 independent experiments.
2. MCL1^BCL2TM^ retains the ability to bind NOXA. *Mcl1^-/-^* MEFs stably expressing HA-NOXA or HA-NOXA^3E^ (the mutant that does not bind MCL1) were engineerd to also express FLAG-MCL1 or FLAG-MCL1^BCL2TM^. Lysates were prepared from cells cultured with proteasome inhibitor MG132 (10 μM for 8 h) and subjected to HA immunoprecipitation.
3. MCL1^BCL2TM^ associates predominantly with the heavy membrane fraction. Protein lysates were prepared from cytosolic (C) and heavy membrane (HM) fractions derived from *Mcl1*^-/-^ MEFs expressing either FLAG-MCL1 or FLAG-MCL1^BCL2TM^.

**Supplementary Table S1. List of sgRNA sequences used in this study.**

**Supplementary Table S2. Indels measured at sgRNA target sites by DNA sequencing.**

Indels were measured by sequencing PCR amplicons encompassing sgRNA target sites and aligning reads to the wild-type locus. In clones where more than two indel sizes were observed the parental cells likely possessed an additional copy (or copies) of the targeted gene. In clones where only one allele size was observed the parental cells may have had only one copy of the gene, or both alleles may have been repaired similarly to generate indels of the same size. No clones for which wild-type sequence remained were used in the study.

**Supplementary Table S3. Quantitation of changes in sgRNA abundance after sorting for GFP^high^ cells and statistical ranking of gene enrichment.**

The table comprises the following elements for each gene represented in the library: the total number of sgRNA targeting that gene in the library, the Log2 fold change for each of those sgRNA (calculated as residuals to the lowess curve fitted to the data in Figure 2b), and the ranked order for the Log2 fold change of each sgRNA. (N, B, n, b) correspond to the parameters that yielded the minimum hypergeometric p-value,^1^ where N is the population size (the total size of the library), B is the number of successes present in the library (sgRNA targeting a particular gene), n is the sample size, and b is the number of successes in the sample. p-values were corrected to false discovery rates (FDR) using the Benjamini-Hochberg method.

**References**

1. Eden E, Lipson D, Yogev S, Yakhini Z. Discovering motifs in ranked lists of DNA sequences. *PLoS Comput Biol* 2007, **3**(3)**:** e39.
